# Ultra-high resolution, 3-dimensional magnetic resonance imaging of the atherosclerotic vessel wall at clinical 7T

**DOI:** 10.1371/journal.pone.0241779

**Published:** 2020-12-14

**Authors:** Martin J. Willemink, Bram F. Coolen, Hadrien Dyvorne, Philip M. Robson, Ilda Bander, Seigo Ishino, Alison Pruzan, Arthi Sridhar, Bei Zhang, Priti Balchandani, Venkatesh Mani, Gustav J. Strijkers, Aart J. Nederveen, Tim Leiner, Zahi A. Fayad, Willem J. M. Mulder, Claudia Calcagno

**Affiliations:** 1 Department of Radiology, University Medical Center Utrecht, Utrecht, The Netherlands; 2 Department of Radiology, Stanford University School of Medicine, Stanford, California, United States of America; 3 Department of Biomedical Engineering and Physics, Amsterdam UMC, University of Amsterdam, Amsterdam, The Netherlands; 4 Department of Radiology and Nuclear Medicine, Amsterdam UMC, University of Amsterdam, Amsterdam, The Netherlands; 5 BioMedical Engineering and Imaging Institute, Icahn School of Medicine at Mount Sinai, New York, New York, United States of America; 6 Department of Radiology, Icahn School of Medicine at Mount Sinai, New York, New York, United States of America; 7 Department of Medical Biochemistry, Academic Medical Centre, University of Amsterdam, Amsterdam, The Netherlands; Monash University, AUSTRALIA

## Abstract

Accurate quantification and characterization of atherosclerotic plaques with MRI requires high spatial resolution acquisitions with excellent image quality. The intrinsically better signal-to-noise ratio (SNR) at high-field clinical 7T compared to the widely employed lower field strengths of 1.5 and 3T may yield significant improvements to vascular MRI. However, 7T atherosclerosis imaging also presents specific challenges, related to local transmit coils and B1 field inhomogeneities, which may overshadow these theoretical gains. We present the development and evaluation of 3D, black-blood, ultra-high resolution vascular MRI on clinical high-field 7T in comparison lower-field 3T. These protocols were applied for *in vivo* imaging of atherosclerotic rabbits, which are often used for development, testing, and validation of translatable cardiovascular MR protocols. Eight atherosclerotic New Zealand White rabbits were imaged on clinical 7T and 3T MRI scanners using 3D, isotropic, high (0.6^3^ mm^3^) and ultra-high (0.4^3^ mm^3^) spatial resolution, black-blood MR sequences with extensive spatial coverage. Following imaging, rabbits were sacrificed for validation using fluorescence imaging and histology. Image quality parameters such as SNR and contrast-to-noise ratio (CNR), as well as morphological and functional plaque measurements (plaque area and permeability) were evaluated at both field strengths. Using the same or comparable imaging parameters, SNR and CNR were in general higher at 7T compared to 3T, with a median (interquartiles) SNR gain of +40.3 (35.3–80.1)%, and a median CNR gain of +68.1 (38.5–95.2)%. Morphological and functional parameters, such as vessel wall area and permeability, were reliably acquired at 7T and correlated significantly with corresponding, widely validated 3T vessel wall MRI measurements. In conclusion, we successfully developed 3D, black-blood, ultra-high spatial resolution vessel wall MRI protocols on a 7T clinical scanner. 7T imaging was in general superior to 3T with respect to image quality, and comparable in terms of plaque area and permeability measurements.

## Introduction

Cardiovascular disease due to atherosclerosis is the main cause of morbidity and mortality worldwide [1]. In the imaging community, there has been much focus in developing and validating non-invasive *in vivo* techniques to characterize arterial vessel wall atherosclerosis and to measure atherosclerotic disease burden non-invasively [2–4]. Specifically, vessel wall measurements by quantitative magnetic resonance imaging (MRI), such as vessel wall area, have already been demonstrated to be good predictors of future cardiovascular events [5–8]. These parameters have also been successfully used as surrogate imaging markers of treatment efficacy for approved and novel anti-atherosclerotic compounds [9–14]. In the long term, non-invasive vascular MR imaging may aid in more efficiently diagnosing and stratifying individuals at high risk for cardiovascular events, as well as monitoring patient treatment response [15, 16].

At present, vessel wall MRI still faces significant challenges [9–12, 17–19] when acquired at the most common clinical field strengths of 1.5 and 3T. MRI of the small arterial wall requires imaging with very high-spatial resolution, for accurate quantification of plaque size and to correctly identify relevant high-risk plaque components, such as lipid rich necrotic core and fibrous cap [16]. Ideally, vascular MRI acquisitions should be 3-dimensional (3D), use isotropic voxels and allow extensive spatial coverage along a whole vessel, while avoiding partial volume artifacts in tortuous, disease-prone areas [20, 21]. In addition, efficient “black blood” preparations, which suppress the bright signal from flowing blood, and perivascular fat signal suppression are needed to ensure optimal contrast and delineation of the arterial wall with respect to the vessel lumen and other neighboring structures [20, 22, 23]. To satisfy all these requirements while maintaining high scan time efficiency, signal-to-noise ratio (SNR) and spatial resolution often need to be compromised when imaging on 1.5 and 3T scanners. This significantly affects the accuracy of atherosclerosis burden measurements and the characterization of plaque composition, especially when using advanced quantitative MR techniques [24]. Particularly, the measurement of functional parameters relevant to high-risk plaque phenotypes, such as enhanced vessel wall permeability and neovascularization using high temporal resolution dynamic contrast enhanced (DCE) MRI [25], may be significantly impacted by low SNR measurements.

The advent of high-field 7T clinical MRI scanners offers new opportunities to surpass these challenges and improve the characterization of at-risk atherosclerotic plaques [26, 27]. In principle, the intrinsically higher SNR at 7T may allow 3D vessel wall imaging with isotropic high spatial resolution, improved image quality and within imaging times suitable for clinical examinations. However, arterial vessel wall MRI at 7T also presents specific issues, for example related to the use of local transmit coils and B1 field inhomogeneities [28], which may overshadow these theoretical advantages over lower field 1.5 and 3T clinical scanners.

In this study, we aimed to assess the advantages and disadvantages of high-field clinical 7T vessel wall MRI for quantification of plaque burden and permeability, in comparison with 3T field strength. 3D vessel-wall black blood MRI protocols developed on a clinical 7T scanner were applied for high and ultra-high spatial resolution *in vivo* imaging of the atherosclerotic rabbit abdominal aorta and benchmarked against equivalent acquisitions at clinical 3T [18, 29]. The rabbit aorta is similar in size to the human coronary arteries [30], and rabbit aortic plaques recapitulate many phenotypical characteristics of human plaques [31]. Since rabbits can be imaged on clinical MR scanners, this relevant animal model of atherosclerosis has been extensively used in the past by our group and others for robust development, testing and validation of cardiovascular MR imaging protocols before translation into humans [17, 32–39].

## Methods

### Ethics statement

This study was conducted after review and approval by the Institutional Animal Care and Use Committee (IACUC) of the Icahn School of Medicine at Mount Sinai.

### Animal model and study design

**Fig 1:**
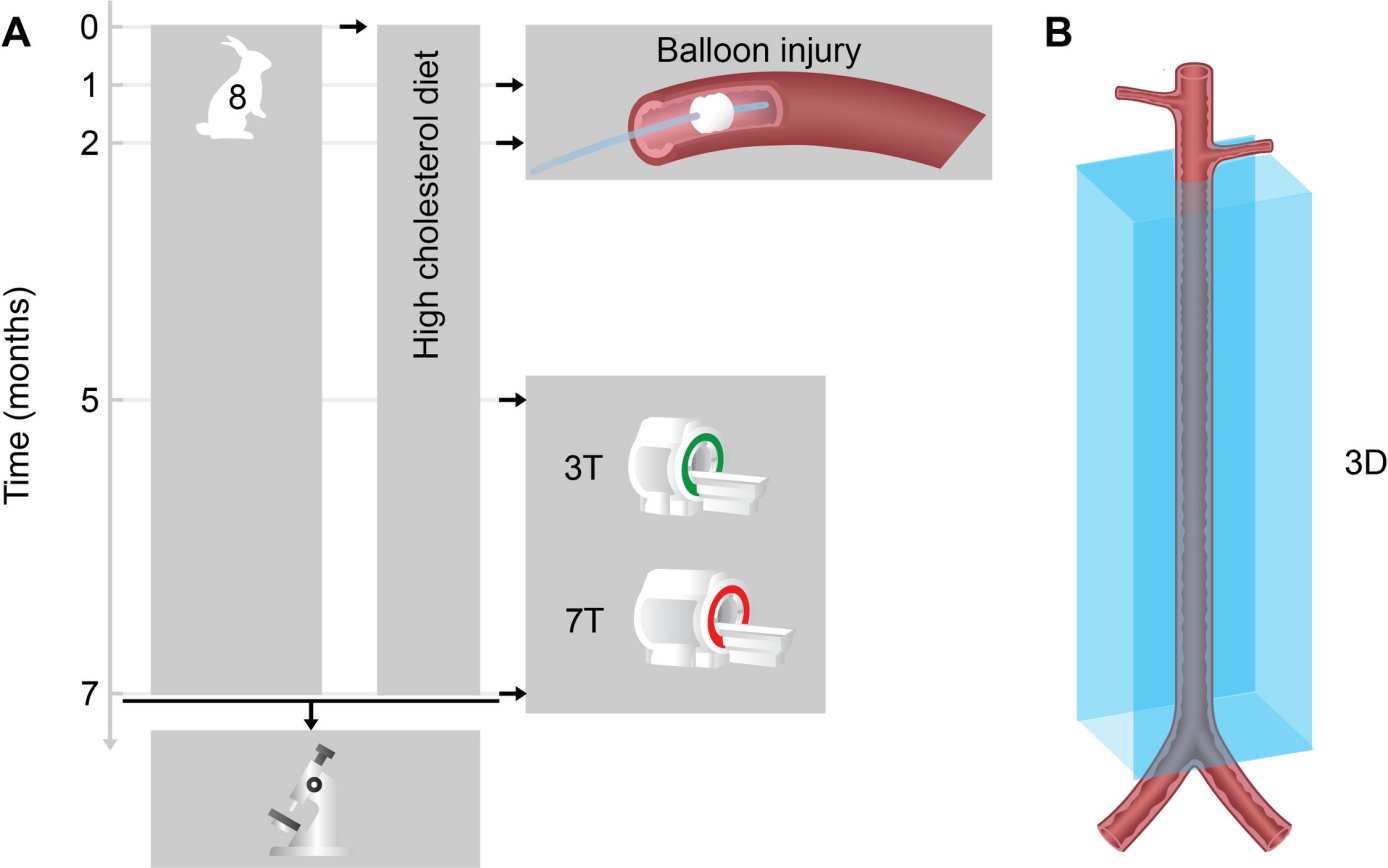
Study design. (A) Atherosclerosis was induced in 8 male *New Zealand White* rabbits. Rabbits were imaged 5–7 months after high-fat diet initiation. 3D imaging sequences were acquired from just below the left renal artery until the aortic bifurcation (B).

Atherosclerosis was induced in 8 male New Zealand White (NZW) rabbits using a previously validated approach [9, 10, 17–19, 29, 40–42], consisting of a combination of high-fat diet and double balloon injury of the infrarenal abdominal aorta. All rabbits were imaged between 5 and 7 months of high-fat diet containing 0.3% cholesterol and 4.7% coconut oil for 2 months and 0.15% and 4.7% coconut oil thereafter. Animals were sacrificed after the last imaging time-point (Fig 1). Animals were imaged twice, once on a 7T clinical scanner (Magnetom, Siemens Healthineers, Erlangen, Germany), and once on a 3T clinical scanner (mMR, Siemens Healthineers, Erlangen, Germany).

### 7T rabbit coil design

**Fig 2:**
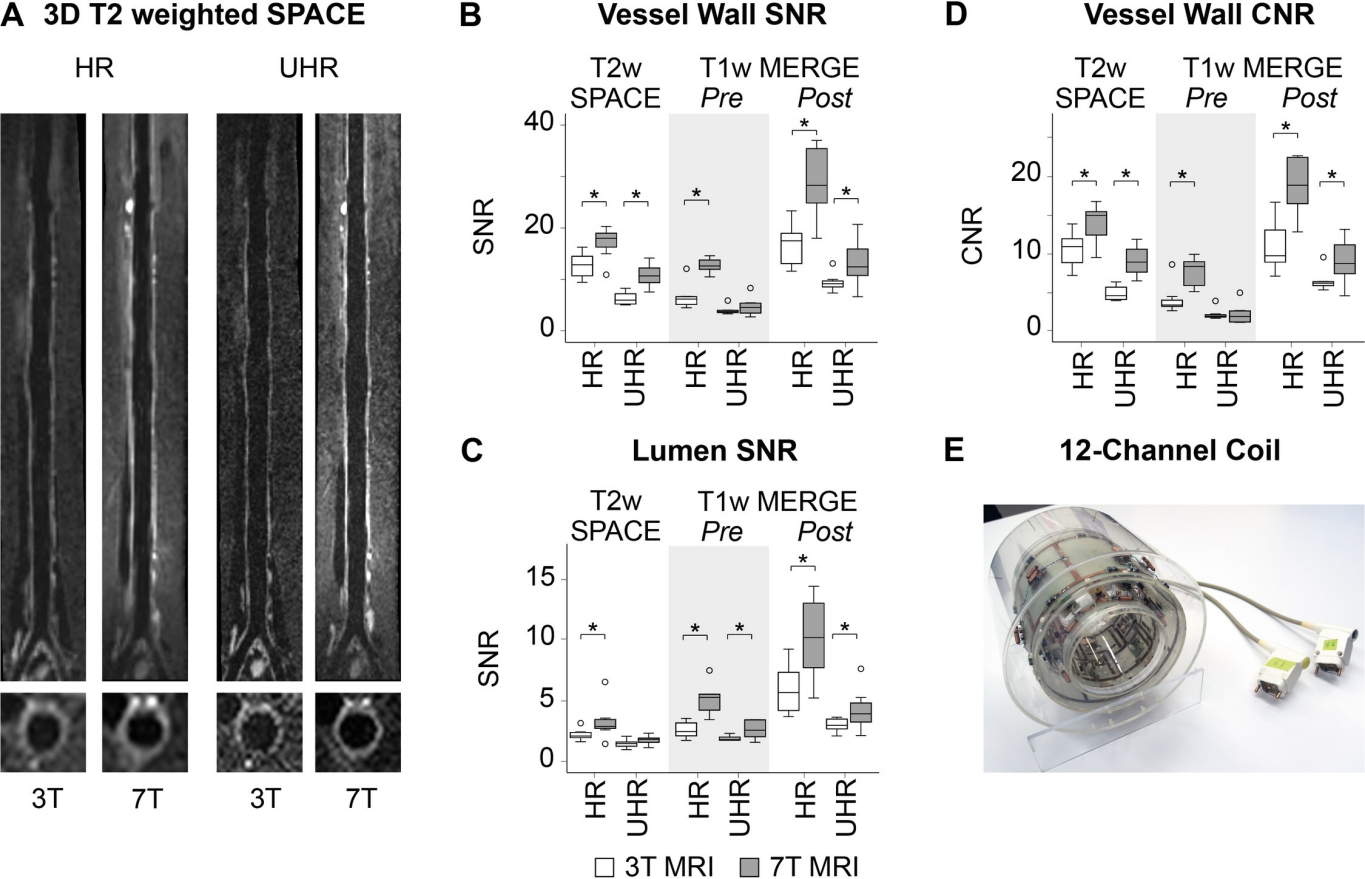
Image quality results. Images of the aorta acquired with 3D T2 weighted SPACE at high resolution (0.6^3^ mm^3^, HR) and ultra-high resolution (0.4^3^ mm^3^, UHR) on a 3T and 7T MRI scanner (A). Boxplots indicating SNR measured in the vessel wall (B) and lumen (C); and CNR, D) for T2w SPACE, and pre-contrast and post-contrast T1w MERGE sequences. The 12-channel receive-only coil is displayed (E). * indicates significantly different based on Wilcoxon-signed ranks tests; SPACE, *Sampling Perfection with Application optimized Contrasts using different flip angle Evolution; MERGE*, *Motion sensitized driven Equilibrium prepared Rapid Gradient Echo; Pre*, *pre-contrast administration; Post*, *post-contrast administration*.

At 7T, a custom-made coil was specifically designed to achieve optimal transmit/receive efficiency along the entire rabbit abdominal aorta for high SNR imaging of the arterial vessel wall. This coil consists of a transmit-only quadrature-driven high-pass birdcage and 12 receive-only loop elements. The transmit birdcage is located on a cylinder with a diameter of 20.3 cm, while the receive elements are located on a cylinder with a diameter of 13.3 cm. The two cylinders are concentric. The 12-channel receive-only array is arranged into two rows in the z direction, with 6 elements in each row (Fig 2E). For the 3T scans a Tx/Rx 15-channel knee coil was used for imaging (Quality Electrodynamics (QED), Mayfield, OH).

### MR image acquisition

On both scanners, localizer scans and time-of-flight (TOF) angiography were used to clearly identify the rabbit abdominal aorta [18, 29]. Subsequently, the following sequences were acquired:

1. 3D T2w SPACE [20, 22] (**S**ampling **P**erfection with **A**pplication optimized **C**ontrasts using different flip angle **E**volution),
2. Pre-contrast 3D T1w MERGE [21] (**M**otion **S**ensitized **D**riven **E**quilibrium (MSDE) [23] prepared **R**apid **G**radient **E**cho),
3. Dynamic contrast enhanced (DCE) MRI using 3D T1w MERGE [18], and
4. Post-contrast 3D T1w MERGE [21].

3D T2w SPACE was used to quantify atherosclerotic plaque burden (defined as vessel wall area), while DCE-MRI and pre/post-contrast 3D T1w MERGE were used to quantify plaque neovascularization/permeability. DCE-MRI was acquired before, during and after manual injection of the T1-shortening MR contrast agent gadolinium (Gd) diethylenetriamine penta-acetic acid (DTPA), at the dose of 0.1 mmol/Kg body weight. At both field strengths, all sequences, with the exception of DCE-MRI, were acquired using 2 different spatial resolutions: 0.6x0.6x0.6 mm^3^ (**High Resolution, ‘HR’**) and 0.4x0.4x0.4 mm^3^ (**Ultra-High Resolution, ‘UHR’**). 3T and 7T acquisition times were 3:50 min for T2SPACE HR and 11:06 min for T2SPACE UHR, while acquisition times of pre- and post-contrast 3D T1w MERGE were 2:39 min for 3T and 7T HR, and 5:46 min for 3T and 7T UHR, respectively. DCE-MRI was imaged only at a resolution of 0.6x0.6x0.6 mm^3^ (**HR**), since only one contrast agent injection was performed per imaging session, and to allow imaging with a sufficiently high temporal resolution for the quantification of plaque permeability [18, 29]. For all sequences, imaging parameters were kept as consistent as possible across field strengths to allow for direct comparison of signal-to-noise (SNR) measurements. No parallel imaging was used. Tables detailing imaging parameters for all sequences at each field strength can be found in the S1 Table.

### MR image analysis

After image acquisition, data was transferred to a workstation with Osirix software for image analysis (http://www.osirix-viewer.com). All images were reformatted in the axial plane before analysis. For all sequences, analyses were performed in axial slices from the left renal artery, down to the aortic bifurcation.

#### Signal to noise (SNR) and contrast to noise (CNR) ratio measurements

Vessel wall signal intensity was calculated by averaging the MR signal in the area defined by the inner and outer vessel wall contours. Noise regions of interest (ROIs) were traced on the 3 middle slices of each sequence, in the native acquisition plane (sagittal). Care was taken to place noise regions in the middle section of the image, where transmit and receive efficiency are higher, and in areas with no artifacts. Standard deviation of noise ROIs, std(noise), was calculated for each slice, averaged across the 3 slices, and then used to calculate SNR assuming Rician distributed noise as follows [43]:

$$SNR=\frac{signal}{1.5∙std(noise)}$$ (1)

Contrast-to-noise ratio (CNR) between vessel wall and lumen was calculated as the normalized difference between vessel wall and lumen SNR. SNR and CNR were calculated for 3D T2w SPACE, and pre and post-contrast 3D T1w MERGE sequences, at both field strengths and spatial resolutions. Slice-by-slice SNR and CNR were averaged for each rabbit from the renal arteries to the iliac bifurcation, and these values were used for statistical analysis. All ROIs were manually placed.

#### Vessel wall area measurements

For each traced axial slice, vessel wall area was calculated from 3D T2w SPACE acquisitions by tracing and subtracting the areas defined by the outer and inner vessel wall contours. Slice-by-slice area measurements were averaged for each rabbit from the renal arteries to the iliac bifurcation, and these values were used for statistical analysis.

#### Dynamic Contrast Enhanced (DCE) MRI

Enhanced vessel wall permeability and increased neovascularization are hallmarks of atherosclerotic plaques at high-risk for causing severe clinical events, such as myocardial infarction and stroke [44]. In the past, our group and others have demonstrated that these features can be reliably quantified using *in vivo* DCE-MRI at 1.5T and 3T, both in patients [45–49] and large animal models [17, 18, 32] of atherosclerosis. This technique consists in the rapid acquisition of images before, during and after the injection of a gadolinium (Gd) based, T1 shortening contrast agent. Here, for DCE-MRI analysis inner and outer vessel wall contours were traced on post-contrast 3D T1w MERGE images. In several rabbit studies we have demonstrated that the area under the contrast agent uptake curve (AUC) from *in vivo* vessel wall DCE-MRI is a good surrogate measure of atherosclerotic plaque permeability [17, 18, 32], as validated by histology [17, 32], and by measuring vessel wall permeability to Evans Blue (EB) dye using near infra-red fluorescence (NIRF) [18]. In line with these historical data, plaque contrast agent uptake was measured by calculating the area-under-the-curve (AUC) of the MRI signal intensity over time, up to 2 minutes after contrast agent injection, averaged in each vessel wall ROI [18]. For statistical analysis, slice-by-slice AUC values were averaged across all analyzed slices, or in segments corresponding to *ex vivo* Evans Blue ROIs (see below for more details).

### Ex vivo Evans Blue (EB) Near Infrared Fluorescence (NIRF) and histology

Within 48 hours from the second imaging session, rabbits were euthanized using an intravenous injection of sodium pentobarbital (100 mg/kg), and immediately perfused using 1L of saline solution. Before euthanasia animals were injected with 6 mL of 0.5% Evans Blue (EB, Sigma Aldrich, St. Louis, MO) in 1x PBS through a 22G catheter placed in a marginal ear vein. For each session, one animal not injected with EB served as control. EB was allowed to circulate for 30 minutes to allow for binding to serum albumin and extravasation in the vessel wall. After euthanasia, rabbit abdominal aortas were harvested, measured in length and imaged with a Xenogen IVIS-200 optical imaging system (Perkin Elmer Inc., Shelton, CT, USA). The excitation and emission filters for Evans Blue were set to 605 and 680 nm, respectively, with an exposure time of 4 s, binning at 8, FOV 12.2 cm. After fluorescence imaging, the abdominal aorta was divided into 12 segments, which were fixed using a 10% buffered formalin solution. The first 4 segments encompassed a 1.6 cm segment (0.4 cm per segment) right below the left renal artery, where formation of atherosclerotic plaques was more prominent. The other 8 segments were equally spaced from the bottom of the 4^th^ section, up to the iliac bifurcation. Within 24 hours from fixation, specimens were embedded into paraffin blocks. Each paraffin block was sectioned (5 μm) onto glass slides and stained using Masson’s trichrome stain for vessel wall area measurements. NIRF images were analyzed by placing square 0.5x0.5 cm^2^ ROIs in the rabbit abdominal aortas from the left renal artery, down to the iliac bifurcation. An ROI of the same size was also placed in a portion of skeletal muscle harvested from each rabbit, acquired within the same FOV and with the same imaging parameters as a control tissue. Average and total radiant efficiency [(p/s/cm^2^/sr)/(μW/cm^2^)] for each ROI were recorded as a measure of EB deposition in the aorta. Outer and inner vessel wall contours were traced on digitized histology slides using Panoramic Viewer software (http://www.3dhistech.com/pannoramic_viewer). Vessel wall area was calculated by subtracting inner from outer vessel wall contour and averaged for each rabbit across the whole aorta (segments 1–12).

### Statistical analysis

Statistical analyses were performed using IBM SPSS version 24.0 (SPSS Inc, Chicago, Illinois, USA) and MedCalc version 16.8.4 (Mariakerke, Belgium). Unless otherwise stated, data are listed as medians (first interquartile–third interquartile). Non-parametric tests were used since all quantified parameter values were not normally distributed. The Wilcoxon signed ranks test was applied to compare dependent data (vessel wall area measurements using imaging and image quality in terms of SNR and CNR) and independent data were assessed with the independent samples Mann-Whitney U test (average and total radiant efficiency on Evans Blue Near Infra-Red Fluorescence and vessel wall area measurements using histology). Correlations between vessel wall area measurements performed on imaging and histology were evaluated using the Spearman’s correlation coefficient with 95% confidence intervals (95% CI). The Spearman’s correlation coefficient was also used for comparison between DCE measurements and EB results. Correlations between all DCE measurements combined for both time points at 3T and 7T were evaluated using the intra-class correlation coefficient (ICC) with 95% CI. These ICC values were calculated for all individual segment measurements as well as for averaged measurements per animal. ICC analyses were also used to compare VWA measured at 3T with 7T. A p-value < 0.05 was considered statistically significant.

## Results

### Image quality: SNR and CNR

At the same spatial resolution, we found SNR to be significantly higher (p<0.05) at 7T compared to 3T for almost all acquisitions, with the exception of vessel wall pre-contrast UHR 3D T1w MERGE (p = 0.093) and lumen UHR 3D T2w SPACE (p = 0.069), where the SNR increase at 7T was not statistically significant. Median SNR gain at 7T with respect to 3T was +40.3 (35.3–80.1)%.

In vessel wall imaging, the CNR indicates the ability to discern the vessel wall from the lumen and the surrounding tissues. CNR was also found to be significantly higher at 7T as compared to 3T, except for vessel wall pre-contrast UHR 3D T1w MERGE (p = 0.889). Median CNR gain was +68.1 (38.5–95.2)%.

Irrespective of field strength, aortic vessel wall MRI SNR and CNR were higher at **HR** (0.6x0.6x0.6 mm^3^ voxel size) as compared to **UHR** (0.4x0.4x0.4 mm^3^) for all acquisitions (p = 0.012 for all comparisons). Our SNR and CNR results are in line with previously published data of the human carotid arteries [26, 27].

Detailed results of image quality measurements SNR and CNR are listed in Table 1 and Fig 2.

**Table 1:** Image quality in terms of signal-to-noise and contrast-to-noise ratios.

Region of interest		Resolution	SNR		SNR Gain		CNR		CNR Gain	
			*3T*	*7T*			*3T*	*7T*		
Vessel wall	T2w SPACE	High	12.9 (11.3–14.3)	18.0 (16.9–18.9)	*+39*.*9%*	***P = 0*.*012***	10.9 (9.3–11.9)	15.0 (12.5–15.4)	*+37*.*2%*	***P = 0*.*012***
		Ultra-high	5.9 (5.3–6.9)	10.7 (9.5–11.5)	*+79*.*8%*	***P = 0*.*012***	4.5 (4.1–5.3)	8.9 (7.7–10.0)	*+95*.*6%*	***P = 0*.*012***
			***P = 0*.*012***	***P = 0*.*012***			***P = 0*.*012***	***P = 0*.*012***		
	Pre-contrast T1w MERGE	High	6.2 (5.3–6.7)	12.6 (12.0–13.4)	*+103*.*2%*	***P = 0*.*012***	3.3 (3.1–3.7)	8.3 (6.1–8.9)	*+150*.*0%*	***P = 0*.*012***
		Ultra-high	3.7 (3.6–4.0)	4.5 (2.7–5.4)	*+21*.*4%*	*P = 0*.*093*	1.9 (1.7–2.0)	1.8 (1.1–2.5)	*-3*.*6%*	*P = 0*.*889*
			***P = 0*.*012***	***P = 0*.*012***			***P = 0*.*012***	***P = 0*.*012***		
	Post-contrast T1w MERGE	High	17.5 (13.3–18.5)	28.3 (19.0–34.8)	*+61*.*2%*	***P = 0*.*012***	9.7 (8.9–12.7)	18.9 (16.6–22.4)	*+93*.*8%*	***P = 0*.*012***
		Ultra-high	9.1 (8.6–9.5)	12.5 (10.9–15.7)	*+36*.*5%*	***P = 0*.*017***	6.1 (5.9–6.3)	8.7 (7.5–11.8)	*+42*.*4%*	***P = 0*.*017***
			***P = 0*.*012***	***P = 0*.*012***			***P = 0*.*012***	***P = 0*.*012***		
Lumen	T2w SPACE	High	2.1 (1.9–2.3)	2.9 (2.7–3.3)	*+38*.*7%*	***P = 0*.*025***				
		Ultra-high	1.5 (1.3–1.6)	1.8 (1.7–1.9)	*+22*.*7%*	*P = 0*.*069*				
			***P = 0*.*012***	***P = 0*.*012***						
	Pre-contrast T1w MERGE	High	2.5 (2.2–3.1)	5.2 (4.5–5.5)	*+110*.*8%*	***P = 0*.*012***				
		Ultra-high	1.8 (1.7–1.9)	2.5 (2.0–3.4)	*+40*.*7%*	***P = 0*.*036***				
			***P = 0*.*017***	***P = 0*.*012***						
	Post-contrast	High	5.6 (4.3–7.0)	10.1 (8.2–12.4)	*+81*.*0%*	***P = 0*.*012***				
	T1w MERGE									
		Ultra-high	2.9 (2.7–3.4)	3.9 (3.5–4.5)	*+31*.*8%*	***P = 0*.*025***				
			***P = 0*.*012***	***P = 0*.*012***						

SNR is evaluated within the lumen and the vessel wall and CNR is evaluated based on vessel wall and lumen measurements. P-values per row indicate comparison between 3T and 7T and P-values per column indicate comparison between high resolution and ultra-high resolution. P-values are based on Wilcoxon-signed ranks tests.

SNR, signal-to-noise ratio; CNR, contrast-to-noise ratio; SPACE, Sampling Perfection with Application optimized Contrasts using different flip angle Evolution; MERGE, Motion Sensitized Driven Equilibrium prepared Rapid Gradient Echo.

### Vessel wall area measurements by 3D T2 weighted SPACE MRI and histology

There were no absolute differences between VWA measurements from **HR** and **UHR** 3D T2w SPACE acquired at either 3T or 7T (p = 0.779 and p = 0.069, respectively). These results are in concordance with data obtained in clinical studies on the carotid arteries using 2D T1w fast gradient echo (FGE) and T2w turbo spin echo (TSE) imaging [27]. As shown by previous studies, the absolute value of quantitative plaque burden measurements depends on the acquired spatial resolution, with lower resolutions resulting in higher measurements [50]. In line with these results, in our study vessel wall area (VWA) measurements were significantly higher for **HR** as compared to **UHR** 3D T2w SPACE, both at 3T and 7T (p = 0.012). Intra-class correlation coefficients between VWA measured with 3T and 7T were 0.873 (95% CI 0.493–0.973) for **HR** and 0.966 (95% CI 0.840–0.993) for **UHR** images.

**Fig 3:**
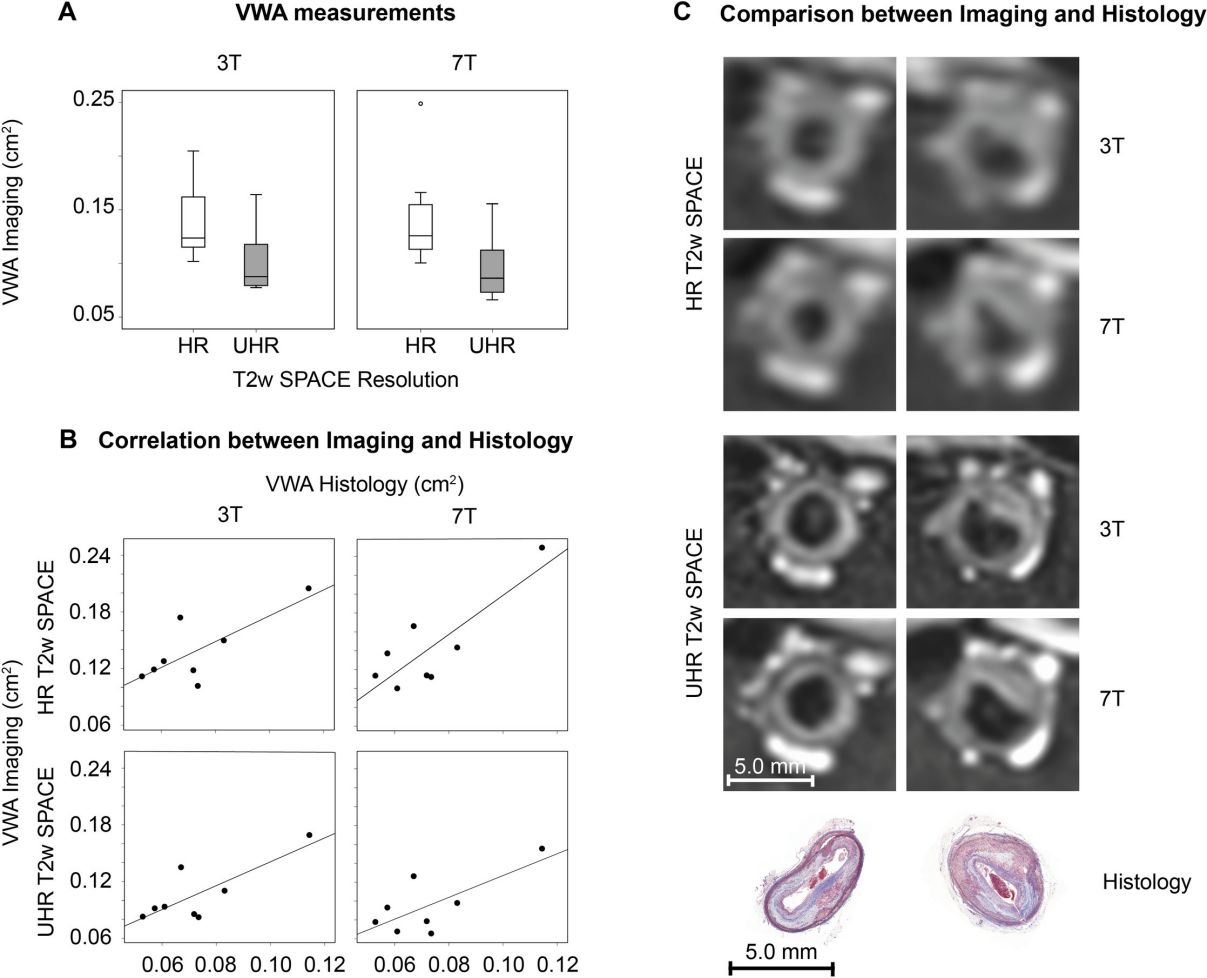
Vessel wall area results. Results of vessel wall area imaging measurements for 3T and 7T (A). Spearman’s correlations between imaging and histology were positive (however not significant) (B). Comparisons of high resolution 3T and 7T images and histology (C) for a plaque with concentric thickening (left) and a larger plaque with eccentric thickening (right). *HR*, *high resolution; UHR*, *ultra-high resolution; VWA*, *vessel wall area*.

Correlations between VWA calculated from imaging and histology were positive but not significant for all spatial resolutions and field strengths with Spearman’s correlation coefficients ranging from 0.405 (95% CI -0.420–0.863, p = 0.320) for UHR 3D T2w SPACE acquired at 7T to 0.476 (95% CI -0.344–0.884, p = 0.233) for HR 3D T2 SPACE acquired at 7T. This result may be due to the fact that MRI and histology were correlated after averaging across the whole vessel, because of intrinsic difficulties in accurately matching corresponding imaging and histological slices. Results of the vessel wall area measurements are listed in Table 2 and displayed in Fig 3.

**Table 2:** Imaging Vessel Wall Area (VWA) measurements.

MR System	VWA (cm^2^)		
	3T	7T	
*High resolution*	0.12 (0.11–0.17)	0.13 (0.11–0.16)	*P = 0*.*779*
*Ultra-high resolution*	0.09 (0.08–0.12)	0.09 (0.07–0.12)	*P = 0*.*069*
	***P = 0*.*012***	***P = 0*.*012***	

VWA measurement results for 3D T2 weighted SPACE acquired on 3T and 7T MR system at high (0.6^3^ mm^3^) and ultra-high (0.4^3^ mm^3^) resolution. VWA measurements at high resolution were significantly larger than ultra-high resolution measurements for both MR systems. VWA values are listed as medians (quartiles). P-values per row indicate comparison between 3T and 7T and P-values per column indicate comparison between high resolution and ultra-high resolution. P-values based on Wilcoxon signed ranks tests.

VWA, vessel wall area; SPACE, Sampling Perfection with Application optimized Contrasts using different flip angle Evolution.

### Vessel wall permeability by in vivo DCE-MRI and ex vivo NIRF imaging

**Fig 4:**
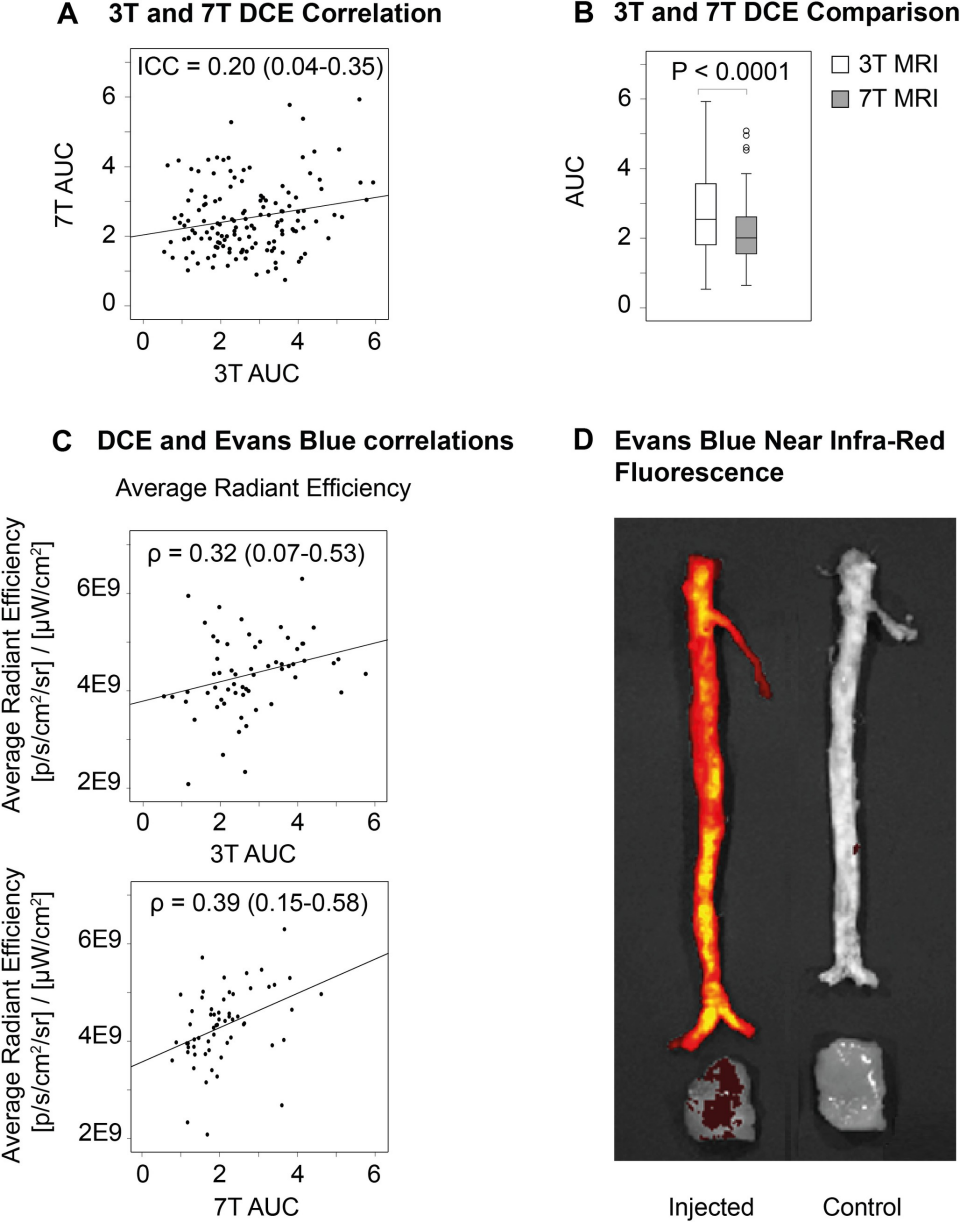
Correlations between 3T and 7T MRI displayed for Dynamic Contrast Enhanced images (DCE) data. (A). Absolute comparison of DCE measurements between 3T and 7T (B). Correlation between Evans blue measurements and DCE measurements (C). Infra-red fluorescence images of two rabbits, one Evans blue injected rabbit and one control rabbit without Evans blue injection (D).

In our study, AUC measurements from 3T and 7T DCE-MRI were positively and significantly correlated, with an ICC of 0.201 [95% CI 0.037–0.354]) (Fig 4), indicating modest concordance between measurements taken at the two different field strengths. In fact, AUC measurements differed significantly between 3T (2.546 [1.810–3.571) and 7T (2.011 [1.552–2.622], p<0.001). However, AUC measurements were positively and significantly correlated with average and total radiant efficiency of *ex vivo* Evans Blue NIRF for both 3T and 7T DCE (Table 3 and Fig 4). Correlations were stronger for 7T (ρ = 0.385 (95% CI 0.146–0.582) for average radiant efficiency and ρ = 0.392 (95% CI 0.153–0.587) for total radiant efficiency) compared to 3T (ρ = 0.321 (95% CI 0.075–0.530) for average radiant efficiency and ρ = 0.291 (95% CI 0.043–0.506) for total radiant efficiency). Average and total radiant efficiency, averaged across all ROIs in a vessel, and across animals, were substantially higher in animals injected with EB (total radiant efficiency 6.43 (5.15–9.24) (p/s/cm^2^/sr)/(μW/cm^2^)) with respect to non-injected, control animals (0.56 (0.55–0.56) (p/s/cm^2^/sr)/(μW/cm^2^).

**Table 3:** Correlations between Evans blue and dynamic contrast enhanced imaging for all measurements.

MRI	EB	Spearman’s correlation		
		*ρ*	*95% CI*	*P-value*
3T	Tot	0.291	(0.043–0.506)	**0.023**
	Ave	0.321	(0.075–0.530)	**0.012**
7T	Tot	0.392	(0.153–0.587)	**0.002**
	Ave	0.385	(0.146–0.582)	**0.002**

P-values based on Spearman’s correlation.

EB, Evans blue; Tot, total radiant efficiency; Ave, average radiant efficiency; 95% CI, 95% confidence interval.

## Discussion

In this manuscript, we describe the development of a 3D black blood atherosclerosis imaging protocol with ultra-high spatial resolution, high SNR and extensive spatial coverage, on a clinical high-field 7T MR scanner.

As a first step, we have chosen to deploy our 3D vessel wall imaging protocol in atherosclerotic rabbits. Assessing the performance of 7T vessel wall imaging in this validated animal model before translation into humans has allowed us to more easily control for possible confounding factors that may render the comparison between different field strengths more challenging. Some of these potential issues are i) bulk motion, which can be avoided by the use of anesthesia in the animal model; ii) the prominent field inhomogeneities apparent in certain relevant human anatomies, such as the neck, where the carotid arteries are located, compared to the rabbit abdomen; and iii) the presence of tortuous vessels in plaque prone areas in humans, such as the carotid bulb, or the intracranial circulation, in comparison with the rabbit infra-renal abdominal aorta.

Because of its specific features, the rabbit model has been extensively used in the past by our groups and others for developing, testing and validating cardiovascular MR imaging protocols before translation into humans [17, 32–39]. Atherosclerotic rabbits are a validated model of cardiovascular disease: the rabbit abdominal aorta is comparable in terms of vessel wall thickness and area to the human coronary arteries [30], and rabbit aortic plaques present many of the characteristic features of high-risk human lesions, such as the presence of active plaque inflammation, abundant plaque macrophages, as well as neovascularization and enhanced vessel wall permeability [51]. While being a small animal model, rabbits can be imaged on clinical MR scanners, and therefore represent an attractive and cost-effective alternative to larger animals for the robust development of protocols that can be readily translated into humans.

Our 7T vascular imaging protocol relies on 3D T2w SPACE [20] and 3D T1w MERGE [21], two validated MR sequences commonly employed for vessel wall imaging to quantify plaque burden and neovascularization/permeability. These sequences were evaluated at the isotropic resolution of 0.6x0.6x0.6 mm^3^ (referred to as “High Resolution”, **HR**), commonly used in our rabbit imaging protocols at 3T [18, 29, 42], and at the higher resolution of 0.4x0.4x0.4 mm^3^ (referred to as “Ultra-High Resolution”, **UHR**). This setup allowed us to evaluate both the effects of spatial resolution and field strength on vessel wall SNR, vessel wall/lumen CNR, and, ultimately, on the quantification of vessel wall area, and plaque neovascularization/permeability.

In our study, we found vessel wall SNR to be overall significantly higher at 7T compared to 3T, with a median SNR gain of +40.3%. This value is considerably lower than the expected theoretical gain of 133% when assuming direct proportionality between SNR and field strength increase. Previous studies employing 2D imaging have also found that vessel wall SNR at 7T is consistently higher than 3T [26, 27], although a higher variability of SNR across subjects at the higher 7T field strength was observed. These and our results may be attributed to the use of different transmit/receive arrays at 3T and 7T, which may significantly impact SNR and image quality regardless of field strength. Other studies, specifically investigating the use of ‘Delay Alternating with Nutation for Tailored Excitation' (DANTE) black blood preparation pulses, found only a modest increase in SNR and CNR at 7T [52]. In this case [52] more prominent aliasing and motion artifacts were observed at 7T, as a result of the prolonged scan time necessary to avoid exceeding specific absorption rate (SAR) limits, which may also have contributed to the observed marginal gain in SNR at this field strength. A lower than theoretical SNR gain at 7T may be also attributable to increased T1 and reduced T2 at this higher field strength, leading to a decrease in SNR at equal acquisition times. While imaging parameters (particularly TR and TE) could be optimized to maximize SNR efficiency, the choice for these parameters is not just dictated by relaxation parameters but also by limitations in total scan time and design of the 3D spin/gradient echo read-out train. In addition, not much literature is available on vessel wall relaxation parameters at 7T. We therefore feel that in keeping the sequence parameters similar between 3T and 7T, the best possible comparison between field strengths has been made.

In concordance with clinical studies on the carotid arteries [27], we found good agreement between vessel wall area measured by T2w SPACE at 3T and 7T, for both **HR** and **UHR** images. As previously shown [50], we found that vessel wall area was higher in **HR** with respect to **UHR** images. Vessel wall measurements by MRI showed a positive correlation with vessel wall area measured by histology, which was, however, not statistically significant. This may be attributed to the fact that vessel wall area by MRI and histological measurements cannot be perfectly slice-matched and therefore were correlated after averaging across the whole vessel. Moreover, we found that VWA measured by MRI was overestimated as compared to histology. This well-known discrepancy is due to the much higher resolution provided by microscopic histological images (μm^2^), compared to the much coarser resolution in vivo MRI (mm^2^). Therefore, while histological images allow for a very accurate quantification of vessel wall area, quantifying plaque burden by MRI carries a margin of error which is directly proportional to image resolution. In fact, as it can be observed in Fig 3, panel B overestimation of vessel wall area by MRI is more prominent at in **HR** versus **UHR** images.

While others have reported the use of contrast enhancement imaging for improved detection of vessel wall lesions in patients with posterior circulation ischemia [53] and of peripheral intracranial segments [54] at 7T, to our knowledge we are the first to report the use of dynamic contrast enhanced (DCE) MRI to quantify neovascularization and permeability in the atherosclerotic vessel wall at this higher field strength. We found good concordance between DCE-MRI results at 3T and 7T, and confirmed a correlation between AUC by DCE-MRI and vessel wall permeability evaluated by *ex vivo* Evans Blue, as previously validated at 3T [18]. Differences in AUC values between the two field strengths may be attributed to expected differences in contrast agent relaxivity at 3 and 7T, and to the method used to convert the MR signal to contrast agent concentration prior to AUC calculation. As previously described and validated, contrast agent concentration was estimated in this study as the relative signal enhancement at each point in time after contrast agent injection [18]. A more accurate quantification of contrast agent concentration, which may eliminate these discrepancies, requires accurate quantification of vessel wall pre-contrast T1 relaxation time with the same spatial coverage and resolution of the sequence used for DCE-MRI [55]. While such a method may certainly lead to a more accurate quantification of plaque enhancement, neovascularization and permeability, 3D T1 mapping of the arterial vessel wall is currently a topic of active investigation and its application in this context was beyond the scope of our study [56, 57].

## Conclusions

In conclusion, we demonstrate the successful development of a 3D black blood MRI protocol to assess plaque burden and neovascularization/permeability in a rabbit animal model of atherosclerosis on a high-field clinical 7T scanner, in comparison with 3T. This approach allows imaging with high spatial resolution and extensive spatial coverage of the rabbit arterial vessel wall, comparable in size to the human coronary arteries, with high SNR and CNR. We find that measures of plaque burden and neovascularization/permeability are comparable between the two field strengths, with SNR and vessel wall/lumen CNR being consistently higher at 7T with respect to 3T.

## Supporting information

S1 Checklist The ARRIVE guidelines 2.0: Author checklist. (PDF)

S1 Table Image acquisition parameters. (DOCX)

S1 File (ZIP)

## List of abbreviations

AUC: Area-under-the-curve
CNR: Contrast-to-noise ratio
DANTE: Delay alternating with nutation for tailored excitation
DCE: Dynamic contrast enhanced
EB: Evans blue
FGE: Fast gradient echo
HR: High resolution
ICC: Intra-class correlation coefficient
MSDE: Motion sensitized driven equilibrium
NIRF: Near infrared fluorescence
NZW: New Zealand white
ROIs: Regions of interest
SAR: Specific absorption rate
SNR: Signal-to-noise ratio
SPACE: Sampling perfection with application optimized contrasts using different flip angle evolution
TOF: Time-of-flight
TSE: Turbo spin echo
UHR: Ultra-high resolution
VWA: Vessel wall area
