# Supplementary material for: Ultra-high resolution, 3-dimensional magnetic resonance imaging of the atherosclerotic vessel wall at clinical 7T
Source: PLoS One. 2020 Dec 14;15(12):e0241779. doi: 10.1371/journal.pone.0241779 (PMC7735577; doi:10.1371/journal.pone.0241779)
Supplement: S1 Table — (DOCX) [file pone.0241779.s002.docx]

**S1 Table**: Image acquisition parameters.

|  | **3D T2W SPACE – Sag** | | **3D T1W MERGE (PRE-POST)** | | **3D T1W MERGE (DCE)** | |
| --- | --- | --- | --- | --- | --- | --- |
|  | 3T  (HR/UHR) | 7T  (HR/UHR) | 3T  (HR/UHR) | 7T  (HR/UHR) | 3T | 7T |
| Slice orientation | Sagittal | | Sagittal | | Sagittal | |
| Number of slabs | 1 | | 1 | | 1 | |
| Phase Encode Direction | A->P | | A->P | | A->P | |
| Phase oversampling (%) | 0 | | 0 | | 0 | |
| Slice oversampling (%) | 20/18.2 | | 10/20 | | 10 | |
| Number of slices (per slab) | 30/44 | | 20/30 | | 20 | |
| FOV read (mm) | 160 | | 120 | | 120 | |
| FOV phase (%) | 62.5 | | 100 | | 100 | |
| Slice thickness (mm) | 0.63/0.42 | | 0.63/0.42 | | 0.63 | |
| TR (ms) | 1600 | | 600 | | 600 | |
| TE (ms) | 115/117 | 114/113 | 5.32/4.82 | 4.73/4.96 | 5.32 | 4.73 |
| Averages | 2 | | 2 | | 1 | |
| Concatenations | 1 | | 1 | | 1 | |
| Excitation | Slab-sel. | | Slab-sel. | | Slab-sel. | |
| Flip angle (deg) | 90 | | 20 | | 20 | |
| Fat Sat | Y(strong) | | Y | | Y | |
| Matrix size | 256/384 | | 192/288 | | 192 | |
| Phase resolution (%) | 100 | | 100 | | 100 | |
| Slice resolution (%) | 100 | | 100 | | 100 | |
| Phase partial Fourier | Allowed | | N | | N | |
| Slice partial Fourier | N | | N | | N | |
| Parallel Imaging (GRAPPA, Ref lines) | N | | N | | Y(2,24) | |
| Reordering |  | | Centric | | Centric | |
| Asymmetric echo |  | | Allowed | | Allowed | |
| Dark blood (thickness, flip angle) | N | | N | | N | |
| Bandwidth (Hz/Px) | 574/383 | | 130 | | 130 | |
| Echo Spacing (ms) | 4.42/6.06 | 4.18/5.46 | 12.4/12 | 11.2/11.9 | 12.4 | 11.2 |
| Shots per slice (segments) |  | |  | | 3(36) | |
| Turbo factor | 81/61 | |  | |  | |
| Slice turbo factor | 1 | |  | |  | |
| Echo trains per slice | 2/4 | |  | |  | |
| Echo train duration | 274/279 | 268/262 |  | |  | |
| RF type | Normal | | Normal | | Normal | |
| Gradient mode | Fast/Fast* | | Normal/Fast | | Normal | |
| RF spoiling |  | | Y | | Y | |
| Phase Enc. Rewinder |  | | Y | | Y | |
| Filters | Image filter (sharp,3,3); Dist corr (2D); Raw Filter; Interp Off | | Image filter (sharp,3,3); Dist corr (2D); Raw Filter; Interp Off | | Ime filter (sharp,3,3); Dist corr (2D); Raw Filter; Interp Off | |
|  |  | |  | |  | |
